# Hepatitis C positive organ transplantation to negative recipients at a multiorgan Canadian transplant centre: ready for prime time

**DOI:** 10.1186/s12876-022-02107-1

**Published:** 2022-01-25

**Authors:** Waleed Alghamdi, Khaled Lotfy, Corinne Weernink, Enad Alsolami, Anthony Jevnikar, Patrick Luke, Anton Skaro, Karim Qumosani, Mayur Brahmania, Paul Marotta, Syed M. Hosseini-Moghaddam, Anouar Teriaky

**Affiliations:** 1 Department of Internal Medicine, Faculty of Medicine, King Abdulaziz University, Jeddah, Saudi Arabia; 2 Division of Gastroenterology, Department of Medicine, Western University, London, ON Canada; 3 Division of Nephrology, Department of Medicine, Western University, London, ON Canada; 4 Department of Internal Medicine, College of Medicine, University of Jeddah, Jeddah, Saudi Arabia; 5 Department of Surgery, Western University, London, ON Canada; 6 Division of Infectious Diseases, Department of Medicine, Western University, London, ON Canada; 7 Multiorgan Transplant Program, London Health Sciences Centre, London, ON Canada; 8 Division of Gastroenterology, Department of Medicine, King Abdulaziz University, Building 10, Second Floor, P.O. Box 55603, Jeddah, 21544 Saudi Arabia

**Keywords:** Hepatitis C, Organ transplantation

## Abstract

**Background:**

Transplantation offers the best survival for patients with end stage organ disease. Transplant of hepatitis C virus (HCV) nucleic acid test (NAT) positive organs into negative recipients is a novel strategy that can expand the donor pool. We aim to evaluate our centre’s experience.

**Methods:**

We preformed a retrospective review of anti-HCV NAT positive and negative organs into negative recipients transplanted over 27 months. Primary outcome was the success rate of eradication of HCV post-transplant. Secondary outcomes were rate of transmission of HCV, treatment adverse events, and graft failure.

**Results:**

33 anti-HCV positive organs were transplanted into negative recipients. 22 (66.7%) were NAT positive. Median recipients age was 49 years (interquartile range [IQR] 44.5–62.0) with the majority being males (57.6%). NAT positive organ transplantations included 16 kidneys, 3 livers, 1 kidney-pancreas, 1 liver-kidney, and 1 heart. The most common HCV genotype was 1a (59.1%). The median time to initiating therapy was 41.5 days. SVR12 was 100% in patients who finished therapy. There were no adverse events with therapy and no graft failure.

**Conclusions:**

Anti-HCV NAT positive organ transplantation into negative recipients is safe with excellent eradication rates and no significant adverse events or graft failure. This would expand donor pool to close the gap between supply and demand.

## Introduction

Organ transplantation is a lifesaving procedure that provides a new lease on life for patients with end stage disease. However, due to supply not meeting demand for organ donation, patients die on the waitlist everyday [1–4]. While living donor donation has long been in practice and machine perfusion strategies are being studied to optimize marginal donors, the gap remains large between the donors needed for recipients on the waiting list [5, 6].

Hepatitis C virus (HCV) has long been a leading cause of liver disease and liver transplantation. The introduction of direct acting antivirals (DAAs) over the last decade has dramatically shifted the treatment paradigm for HCV. This has led to a substantial increase in cure rates in excess of 95% which in turn has led to a significant decrease in liver transplantation for HCV. Treatment with DAAs is safe and well tolerated with minimal adverse events and drug-drug interactions. Patients can be treated effectively pre and post-transplant. While HCV post-transplant historically led to inferior graft and patient survival in relation to other liver diseases, this has changed with the DAAs and is no longer an issue [7–9].

The opioid epidemic has led to devastating consequences in North America over the last decade with millions of people with opioid use disorders and tens of thousands of deaths occurring annually. Concomitantly, there has been an exponential increase in the number of new HCV cases over the past decade due, in part, to this and the use of increased risk donors (IRD) primarily from overdose deaths which has also increased over the same period [10–12]. A donor is denoted as IRD based on demographics and behaviors that could increase the risk of certain infections such as HCV, hepatitis B virus (HBV), and human immunodeficiency virus (HIV) [13]. Nucleic acid testing (NAT) has allowed us to rapidly assess the risk of donor derived infections and use organs that may have been otherwise discarded [14].

The prevalence of anti-HCV positive donors is 8.2% among potential donors and more than 30% among those dying of drug overdose. Approximately, 4% of potential donors are anti-HCV NAT positive at the time of donation. Anti-HCV NAT positive donors are viremic while NAT negative donors are not. Anti-HCV NAT negative donors have a risk of contracting HCV viremia in the recipient of less than 1% while NAT positive donors have essentially a 100% risk of viremia. Most of these donors tend to be young with minimal comorbidities. The advent of DAAs has created an opportunity to expand the donor pool further by using these quality organs that would otherwise be discarded for which HCV can be easily cured post-transplant [8, 12, 13, 15].

Anti-HCV NAT positive organ transplantation into negative recipients is a novel strategy that has the capacity to increase the donor pool, improve the quality of transplanted organs, and decrease the waiting time on the transplant list. Clinical trials that have been conducted to date have shown promising results. In this study we provide real world data on our multiorgan transplant centre experience in transplanting anti-HCV NAT negative and NAT positive organs into negative recipients and their outcomes.

## Methods

This is a single centre retrospective study performed in a multiorgan transplant program (MOTP) at London Health Sciences Centre (LHSC) in London, Ontario, Canada between January 1, 2018 and March 31, 2020. Approval for this study was obtained from the Western University Research Ethics Board (REB). All adult organ transplant recipients that received an anti-HCV organ were included in this study. Patients with positive HCV viral loads pre-transplant were excluded. All organ recipients were offered the ability to accept an anti-HCV organ during the initial transplant assessment and educated on the potential benefits as well as risks. They had the option to opt out at any point. Informed written consent was obtained from patients for an IRD donor immediately prior to the transplant. Both donors with neurological determination of death (NDD) and donors with circulatory determination of death (DCD) were included in this study. Donor and recipient demographic variables were collected for this study.

Both anti-HCV NAT negative and NAT positive organs were used. All these organs were IRD. The age cut off for utilization of NAT positive organs was ≤ 45 years. All patients were followed by a hepatologist and infectious disease specialist post-transplant. Post-transplant viral load and serological testing were performed as per our developed protocol for IRD and can be seen in Table 1. This included NAT testing and serology for HCV, HBV, and HIV. For patients that developed viremia to HCV, the genotype was determined and they were started on treatment with DAAs at the discretion of the hepatologist/infectious disease specialist after approval by insurance plans. Once the patient was started on DAAs, HCV PCR testing was performed at end of treatment and to confirm sustained virologic response at 4 weeks (SVR4), 12 weeks (SVR12), and 24 weeks (SVR24) post treatment. Eradication of HCV was defined as a negative serum HCV RNA at week 12 post treatment (SVR12). The immunosuppression protocol was adjusted by the primary team as appropriate.

**Table 1:** LHSC MOTP protocol for IRD organ recipient testing [13]

Organ status	Post-transplant test	Timing of the test
NAT+	HCV NAT	Weekly up to 4 weeks Monthly up to 3 months
	HIV NAT Anti-HBc and HBsAg ($\pm$ HBV NAT)	At 1 month At 3 months
	Anti-HBs, Anti-HBc, and HBsAg	At 12 months
NAT−	HIV NAT and HIV serology HCV NAT and HCV serology Anti-HBc and HBsAg ($\pm$ HBV NAT)	At 1 month At 3 months
	Anti-HBs, Anti-HBc, and HBsAg	At 12 months

*LHSC* London Health Sciences Centre, *MOTP* multiorgan transplant program, *IRD* increased risk donor, *NAT* nucleic acid testing, *HCV* hepatitis C virus, *HIV* human immunodeficiency virus, *HBV* hepatitis B virus, *Anti-HBc* hepatitis B core antibody, *HBsAg* hepatitis B surface antigen, *Anti-HBs* hepatitis B surface antibody

The primary outcome of this study was the rate of eradication of HCV post organ transplantation in viremic patients. Secondary outcomes included rate of viremia of HCV in NAT positive and NAT negative donors, treatment adverse events, and graft failure due to HCV. Data were analyzed using SPSS software, version 26.0 (IBM). We used medians and interquartile range to summarize continuous variables while categorical variables were described using proportions.

## Results

There was a total of 33 IRD organ transplantations included in our analysis. As shown in Table 2, these included 24 (72.7%) kidneys, 5 (15.2%) livers, 2 (6.1%) kidney-pancreas, 1 (3%) simultaneous liver kidney and 1 (3%) heart transplantation. Of the anti-HCV donors, 22 (66.7%) were NAT positive and 11 (33.3%) with NAT negative.

**Table 2:** Baseline characteristics of the anti-HCV NAT positive and NAT negative transplanted organs

Organ Type	Anti-HCV NAT positive organs (N = 22)	Anti-HCV NAT negative organs (N = 11)
Liver	3 (13.6)	2 (18.2)
Kidney	16 (72.7)	8 (72.7)
Kidney-pancreas	1 (4.5)	1 (9.1)
SLK*	1 (4.5)	0 (0)
Heart	1 (4.5)	0 (0)

Data are number of patients (%)

*SLK simultaneous liver kidney transplantation

DCD grafts were 22.7% of anti-HCV NAT positive donors and 45.5% of anti-HCV NAT negative donors. The median kidney donor profile index (KDPI) in anti-HCV NAT positive donors was 39% (IQR 27 to 51%) and 54% (IQR 34 to 90%) in anti-HCV NAT negative donors for kidney transplants. The median donor risk index (DRI) in anti-HCV NAT positive donors was 1.3 (IQR 1.2 to 2.1) and 1.4 (IQR 1.1 to 1.6) in anti-HCV NAT negative donors for liver transplants. A lower KDPI or DRI has better post-transplant graft survival.

As shown in Table 3, at the time of transplantation, the median age of the recipients was 49 years (IQR 44.5 to 62.0) and the majority were males 19 (57.6%). The most common causes of end stage renal disease were diabetes and hypertension while the most common cause of chronic liver disease was alcohol. Of the 11 anti-HCV NAT negative organs transplanted, none of the recipients developed viremia after 3 months of follow up, while all of the anti-HCV NAT positive organ recipients developed viremia.

**Table 3:** Baseline characteristics of anti-HCV NAT positive and NAT negative transplant recipients

Recipient characteristics	Anti-HCV NAT positive organs (N = 22)	Anti-HCV NAT negative organs (N = 11)
Age (years)	47.5 (42.3–62.0)	56 (47–65.0)
Male gender (%)	13 (59.1)	6 (54.5)
Etiology of transplant		
A. Kidney (%)		
Diabetes	4 (18.2)	1 (9.1)
Hypertension	1 (4.5)	4 (36.4)
PCKD	3 (13.6)	1 (9.1)
Nephrotic syndrome	1 (4.5)	1 (9.1)
GN	2 (9.1)	0 (0)
IgA nephropathy	2 (9.1)	0 (0)
Light chain deposition disease	1 (4.5)	0 (0)
Vasculitis	0 (0)	2 (18.2)
Alport syndrome	1 (4.5)	0 (0)
Reflux nephropathy	1 (4.5)	0 (0)
ATN	1 (4.5)	0 (0)
NYD	1 (4.5)	0 (0)
B. Liver (%)		
ASH	1 (4.5)	2 (18.2)
HCV	1 (4.5)	0 (0)
Hepatocellular carcinoma	2 (9.1)	0 (0)
C. Heart (%)		
Ischemic cardiomyopathy	1 (4.5)	0 (0)
CMV (D+/R−) (%) Mismatch	4 (18.2)	3 (27.3)

Data are number of patients (%) or median (IQR)

*GN* glomerulonephritis, *PCKD* polycystic kidney disease, *IgA* immunoglobulin A, *ATN* acute tubular necrosis, *NYD*,not yet determined, *ASH* alcoholic steatohepatitis, *CMV* cytomegalovirus, *D* donor, *R*,recipient

^*^Both recipients were treated for HCV (SVR 12 achieved) and were HCV NAT negative before transplant

There were 2 patients that had been treated and cured for HCV pre-transplant that were reinfected with an anti-HCV NAT positive organ and were retreated post-transplant with achieving eradication. Two donors and three recipients were positive for HBcAb. Treatment prophylaxis was started when necessary post-transplantation based on recipient risk of occult HBV infection and our institution’s protocol for follow up and management of these patients. None of the transplant recipients developed an HBV or HIV infection.

The most common HCV genotype was genotype 1a in viremic patients and the majority of the patients developed HCV viremia by the first week (Table 4). The median time from transplant to starting DAAs therapy was 41.5 days (IQR 31.0 to 54.5). Sofosbuvir/velpatasvir was the most commonly used DAA followed by glecaprevir/pibrentasvir. All viremic patients that completed treatment with DAAs achieved SVR4 and SVR12. Regardless of type of organ transplanted, eradication was achieved in all organ groups.

**Table 4:** Characteristics of donor-derived HCV infection in transplant recipients

Characteristics	Total (N = 22)
HCV genotype (%)	
1a	13 (59.1)
1b	2 (9.1)
2	1 (4.5)
3	6 (27.3)
Detection HCV viral load (IU/mL)	8.9 × 10^5^ (2.4 × 10^5^—5.9 × 10^6^)
HCV viremia detection interval (%)	
Week 1	17 (77.3)
Week 2	3 (13.6)
Week 3	2 (9.1)
Time to start DAA therapy (days)	41.5 (31.0–54.5)
DAA therapy started (%)	22 (100)
DAA therapy (%)	
Sofosbuvir/velpatasvir	10 (45.5)
Glecaprevir/pibrentasvir	9 (40.9)
Ledipasvir/sofosbuvir	2 (9.1)
Elbasvir/grazoprevir	1 (4.5)
SVR4 (%)	21 (95.5)
SVR12 (%)	21 (95.5)*
Adverse events (%)	0 (0)
Graft failure (%)	0 (0)

Data are number of patients (%) or median (IQR)

All patients were treated with 12 weeks of antivirals except for patients with glecaprevir/pibrentasvir that received 8 weeks

^*^All patients who completed their DAA regimen achieved SVR12 (N = 21). One patient died while on Glecaprevir/Pibrentasvir before finishing the course

The median wait time on dialysis pre-transplant for anti-HCV NAT positive renal transplant recipients was 718 days (IQR 512 to 844) and for non-HCV renal transplant recipients was 940 days (IQR 600 to 1386). The median wait time for anti-HCV NAT positive liver transplant recipients was 54 days (IQR 9 to 150) and for non-HCV liver transplant recipients was 106 days (IQR 17 to 224).

There were no significant adverse events that the patients experienced related to DAAs therapy. There was no evidence of graft rejection, graft failure, or fibrosing cholestatic hepatitis related to DAAs or HCV (Table 5). There were no significant drug-drug interactions noted in relation to DAAs and anti-rejection regimens. There were 28 patients (84.8%) that received induction immunosuppression of which 9 (27.3%) received anti-thymocyte globulin and 19 (57.6%) received basiliximab.

**Table 5:** Liver functions of the transplant recipients at baseline and 3 months post transplantation

Recipient characteristics	NAT positive organs (N = 22)	NAT negative organs (N = 11)	Total (N = 33)
Pre-transplantation			
Creatinine (μmol/L)	647 (205.0–1018.0)	614 (305.0–807.0)	614 (230.5–942.0)
Albumin (g/L)	43.5 (38.5–45.5)	40 (38.0–44.0)	43 (38.0–45.0)
Total bilirubin (μmol/L)	7.6 (5.3–10.9)	5.9 (4.4–9.7)	6.5 (4.7–10.5)
INR	1.1 (1.0–1.1)	1.1 (1.0–2.4)	1.1 (1.0–1.2)
ALT (U/L)	16.5 (11.0–20.8)	17 (9.0–31.0)	17 (11.0–21.5)
3 months post-transplantation			
Creatinine (μmol/L)	86 (72.5–117.0)	114.5 (99.5–186.8)	89 (73.0–119.0)
Albumin (g/L)	41 (35.5–44.0)	41 (35.8–43.8)	41 (35.5–44.0)
Total bilirubin (μmol/L)	7 (4.0–9.6)	5.4 (4.2–7.9)	6.9 (4.2–8-9)
INR	1 (1.0–1.2)	1.1 (1.0–1.3)	1 (1.0–1.2)
ALT (U/L)	19 (13.5–34.5)	18 (14.5–27.0)	19 (14.0–29.0)

Data are median (IQR)

*INR* international normalized ratio, *ALT* alanine aminotransferase

One patient with metabolic syndrome that achieved SVR12 had persistently elevated hepatocellular liver enzymes even after cure. This patient had long standing NAFLD pre-transplant and post-transplant liver biopsy confirmed NASH with F2 fibrosis as the cause of liver enzyme elevation. One patient experienced a complicated and prolonged hospital course due to frailty with recurrent sepsis and eventually died from invasive pulmonary aspergillosis.

## Discussion

This study further consolidates the emerging evidence that anti-HCV NAT negative and positive organs can be transplanted into negative recipients effectively and safely. While thousands of organ transplants are performed annually, many patients remain on the waitlist and a substantial amount will die waiting [16, 17]. This has generated significant demand for novel strategies to broaden the donor pool. The catastrophic opioid crisis has led to a substantial increase in anti-HCV NAT negative and positive organs that can be transplanted into negative recipients. Our study provides a real world Canadian multiorgan transplant experience that shows that this can be successfully done outside of a research study.

There were 11 patients that received anti-HCV NAT negative organs in this study and none of these patients developed a donor derived HCV infection after 3 months of follow up post-transplant. The risk of a donor derived HCV infection in the setting of an IRD is variable depending on the risk category of the donor. The risk of HCV infection during the window period usually ranges from 1.4 to 40.8 per 10,000 donors by ELISA and NAT [13]. All 22 patients that were transplanted with an anti-HCV NAT positive organ developed viremia and this is consistent with prior studies where essentially all recipients transplanted with anti-HCV NAT positive organ developed a donor derived HCV infection [18–22]. This study demonstrated some of the benefits of transplanting anti-HCV NAT positive organs into negative recipients. It expanded the donor pool at our centre, led to shortened wait times for organ transplantation, and the transplantation of organs with better predicted graft survival as manifested by lower KDPI and DRI.

We did not encounter any significant difficulties in obtaining insurance coverage for all patients in our cohort. While there were some minor delays in obtaining approval for coverage, the median time to treatment start was 42 days. We did not see any cases of fibrosing cholestatic hepatitis in our patient cohort even in cases where treatment may have been delayed. However, the pre-transplant patient with NAFLD receiving a kidney transplant that was treated and cured for HCV with persistent hepatocellular elevation was found to have NASH with moderate fibrosis on liver biopsy due to NASH. We did not see any graft failure due to HCV in any of the patients with donor derived HCV infections. One comorbid patient had a prolonged intensive care course with enteral feeds that limited treatment initiation. They were eventually started on treatment, but would die due to invasive fungal infection. This highlights the need for pre-transplant careful selection of the candidates for this novel approach.

All patients that developed a donor derived HCV infection from an anti-HCV NAT positive organ were treated with DAAs. The most commonly used DAAs were the pangenotypic agents sofosbuvir/velpatasivir and glecaprevir/pibrentasvir. Therapy was based on several factors including insurance coverage, genotype, and creatinine clearance at time of treatment. The SVR12 rate was 100% in all patients that were treated with DAAs that completed treatment and this is consistent with prior studies. There were no significant adverse events related to treatment. We did not encounter any significant alterations to the immunosuppression regimens while on treatment or any drug-drug interactions requiring medication adjustment. There were no differences in achieving HCV eradication in those that received or did not receive a T cell depleting agent. While the cost of treatment for DAAs can be expensive, transplanting patients using anti-HCV NAT positive organs has already been shown to be cost effective [23].

The organs transplantations in this study were diverse and included kidneys, livers, SLK, kidney pancreas and a heart. In addition to this we transplanted 2 recipients that had undergone treatment and cure for HCV pre-transplant with livers from NAT positive organs and we were successful in curing them again after reinfecting them with HCV. None of the patients in this study developed any HBV or HIV infection. We did not encounter any reactivation of HBV in HBcAb patients that were treated for HCV. HBV prophylaxis was utilized where appropriate specifically in liver transplants as per our institution protocol.

The treatment of donor derived HCV infection in the post-transplant setting can be accomplished by either a delayed or prophylactic approach. Viremia is clearly demonstrated in the delayed approach with treatment started thereafter whereas patients are empirically treated before viremia is manifested in the prophylactic approach. In this study we used a delayed approach as insurance providers required determination of viremia before approving therapy. There are merits to a prophylactic approach as treatment duration can be shorter and starting treatment immediately may limit the risk of fibrosing cholestatic hepatitis. However, complex patients might not be able to undergo treatment immediately after transplant [24–30]. Insurance providers will need to approve treatment pre-transplant for this approach to be more practically utilized outside of a research study. It is imperative to commit to treatment once viremia occurs.

Limitations of this study include the small sample size, being a single centre experience, the potential for bias, and the lack of long-term follow up.

## Conclusions

Our study was able to show some important findings. Anti-HCV NAT negative and NAT positive organs can be successfully and safely transplanted into NAT negative recipients in a real-world experience at a multiorgan transplant program. There has to be a commitment to treatment once a donor derived HCV infection has occurred. Treatment was well tolerated with no significant adverse events and excellent cure rates. Future directions should include large multicentre cohort studies focusing on long term outcomes of this approach.

## Data Availability

The data that support the findings of this study are available on reasonable request from the corresponding author. The data are not publicly available due to privacy or ethical restrictions.

## Abbreviations

HCV: Hepatitis C virus
DAAs: Direct acting antivirals
IRD: Increased risk donors
HBV: Hepatitis B virus
HIV: Human immunodeficiency virus
NAT: Nucleic acid testing
MOTP: Multiorgan transplant program
LHSC: London Health Sciences Centre
REB: Research Ethics Board
SVR4: Sustained virologic response at 4 weeks
SVR12: Sustained virologic response at 12 weeks
SVR24: Sustained virologic response at 24 weeks
SPSS: Statistical Package for the Social Sciences
IBM: International Business Machines Corporation
DCD: Donation after circulatory death
IQR: Interquartile range
HBcAb: Hepatitis B core antibody
NAFLD: Non-alcoholic fatty liver disease
NASH: Non-alcoholic steatohepatitis
ELISA: Enzyme-linked immunosorbent assay
SLK: Simultaneous liver kidney transplantation
Anti-HBc: Hepatitis B core antibody
HBsAg: Hepatitis B surface antigen
Anti-HBs: Hepatitis B surface antibody
BMI: Body mass index
KDRI: Kidney donor risk index
KDPI: Kidney donor profile index
Liver DRI: Liver donor risk index
GN: Glomerulonephritis
PCKD: Polycystic kidney disease
IgA: Immunoglobulin A
ATN: Acute tubular necrosis
NYD: Not yet determined
ASH: Alcoholic steatohepatitis
CMV: Cytomegalovirus
D: Donor
R: Recipient
INR: International normalized ratio
ALT: Alanine aminotransferase
